# Evidence-Supported HBO Therapy in Femoral Head Necrosis: A Systematic Review and Meta-Analysis

**DOI:** 10.3390/ijerph18062888

**Published:** 2021-03-12

**Authors:** Emma Paderno, Vincenzo Zanon, Giuliano Vezzani, Tommaso Antonio Giacon, Thomas L. Bernasek, Enrico M. Camporesi, Gerardo Bosco

**Affiliations:** 1 Environmental and Respiratory Physiology Lab and II Level Master in Diving and Hyperbaric Medicine, Department of Biomedical Sciences, University of Padova, 35122 Padova, Italy; emma.paderno@gmail.com (E.P.); giuliano.vezzani@alice.it (G.V.); gerardo.bosco@unipd.it (G.B.); 2 DHMU at ICCB, Istituti Ospedalieri Bresciani, GSD—University and Research Hospitals, 25128 Brescia, Italy; 3 Adult Reconstruction, Florida Orthopaedic Institute, Tampa, FL 33625, USA; buckteeth@aol.com; 4 TEAM Health Research Institute, Tampa, FL 33606, USA; enrico_camporesi@teamhealth.com

**Keywords:** femoral head necrosis, hyperbaric oxygen therapy, necrosis, osteonecrosis, oxygen, microcirculation, pain

## Abstract

Although many studies have shown that hyperbaric oxygen (HBO) therapy can significantly improve symptoms and quality of life of patients affected by femoral head necrosis, this therapy is not worldwide approved yet. This meta-analysis was performed to evaluate its clinical effect. Relevant studies published before May 2020 were systematically searched using terms related to HBO and femoral head necrosis. Fixed and random-effects models were used to estimate the odds ratio (OR) with 95% confidence intervals (CI). Subgroup analyses and publication bias tests were carried out to explore potential study heterogeneity and bias. Ten studies involving 353 controls and 368 HBO-treated cases were included, most of which were conducted on Asian population. The clinical effect in the HBO therapy group was 3.84 times higher than in the control group (OR = 3.84, 95% CI (2.10, 7.02), *p* < 0.00001). Subgroup analyses showed that the clinical effect of HBO therapy was statistically significant in the Asian subpopulation which represented most of the subjects (OR = 3.53, 95% CI (1.87, 6.64), *p* < 0.00001), but not in the non-Asian subpopulation, probably because of insufficient numerosity (OR = 7.41, 95% CI (0.73, 75.71), *p* = 0.09). The results of this meta-analysis suggest that patients with femoral head necrosis treated with HBO therapy can achieve a significant clinical improvement.

## 1. Introduction

Femoral head necrosis (FHN) [1]—also named avascular necrosis, atraumatic necrosis, or osteonecrosis of the femoral head—is a multifactorial disease [1,2,3,4,5] mainly caused by a compromised blood supply to the bone structure resulting in significant clinical morbidity.

In the United States, 10,000 to 20,000 new cases of FHN are diagnosed each year. The prevalence of FHN worldwide is 300,000 to 600,000 cases [6], and it is increasing. It is unclear whether this is a real increase or a consequence of better awareness and diagnostic advancements [7,8,9,10,11,12].

The etiology and pathophysiology of FHN are not fully understood yet. FHN can result from both traumatic and atraumatic factors, and it is characterized by the death of bone marrow cells and osteocytes. The disease’s initial stages are usually asymptomatic or marked by mild pain radiated to the knee or ipsilateral buttock or by a limited range of motion at the hip and stabbing pain, especially during a forced internal rotation [13]. A detailed interview can identify any eventually associated risk factors through the patient’s medical history. FHN should be considered if patients feel pain in the hips and possess no risk factors in their medical history [14]. If untreated, the disease progresses to a painful limitation of the hip’s active and passive motion until joint collapse. Indeed, FHN is responsible for 5% to 18% of all hip replacements performed [13,14,15].

FHN generally affects middle-aged adults (35–50 years), more often male (4:1/M:F), and bilaterally, therefore patients with a history of previous necrosis must be observed for bilateral FHN [13]. Due to the age group involved, this pathology also has several socio-economic implications [16]. Indeed, sequelae of untreated FHN include femoral head collapse, hip joint degenerative lesions, and subsequent long-term disability [17].

Many studies have shown that one of the essential factors that cause bone cell apoptosis in FHN is insufficient blood supply resulting in fractures, the articular surface’s collapse, and femoral head functional loss. Microscopically, FHN has a typical cellular necrosis pattern, bone resorption, and neoformation with progressive articular surface collapse. Pathologic analysis shows interstitial marrow necrosis of both hematopoietic and adipocyte cells with concomitant interstitial marrow edema. Although osteocytes necrosis occurs after approximately two to three hours of anoxia, histological signs are visible 24 to 72 h following oxygen deprivation [17,18,19]. Reactive hyperemia and capillary revascularization occur in the periphery of the necrotic area. A repair process begins when bone resorption and production incompletely replace the dead bone with living bone [18]. Dead bone is partially resorbed, and new living bone is laminated over dead trabeculae. Bone resorption generally exceeds bone formation in the subchondral trabeculae [17,20,21,22].

For this reason, excessive bone removal leads to loss of structural integrity of trabeculae, subchondral fracture, and joint incongruity [23]. The best pathophysiological models suggest a common pathway with compromised subchondral microcirculation and resultant ischemia [18]. Regardless of the origin of a first insult, an alteration of the osteocytes’ segmental blood flow and death is recognized as the first moment of the femur’s cephalic necrosis. The reduction of vascularization can have an intraluminal or extraluminal origin [24]. A significant decrease in blood flow would be expected to reduce the intraosseous partial pressure of oxygen (pO_2_); assuming a constant oxygen consumption rate, pO_2_ is lower in bone with histological necrosis (mean 44 mmHg) than in bone without it (mean 71 mmHg) [17,18,25].

Three main pathogenic mechanisms lead to decreased blood flow to the head of the femur that may result in ischemia and, consequently, FHN [26,27]:

Vascular interruption by intracapsular fracture or dislocation of the femoral neck (direct trauma to vessels that supply the subchondral bone) [17]; endoluminal obstruction (sickle cell aggregations, clots or lipid thrombi, genetic defect and coagulation factor aberrations and repeated vascular insults patients suffered throughout their lives) [28,29]; elevated pressure within the intraosseous extravascular space compressed by lipocyte hypertrophy or Gaucher cell growth [26]. The increase is often associated with corticosteroid use or chronic alcohol consumption [30,31,32,33,34,35,36,37,38,39] and in Gaucher disease [40]. Another physiopathological mechanism is under investigation: extra-osseous venous obstruction. Although the extra-osseous veins’ impairment happens, there is still uncertainty whether it is a cause or effect [17,27].

Occurrences of FHN have been reported in individuals undergoing radiation, receiving bone marrow transplants, or suffering from metastatic malignancies. Many other risk factors are described, such as smoking, previous hip injury or surgery on that joint, or low bone mass [41]. Other potential causes and conditions include hyperlipidemia, hyperuricemia, pancreatitis, leukemia or lymphoma, hypertriglyceridemia, baropathies, sarcoidosis, HIV/AIDS, Systemic Lupus Erythematosus (SLE), and sickle cell anemia, though the mechanisms behind these are less understood [4,5,18,42].

Early diagnosis of FHN is necessary since clinical success is strictly related to the treatment stage. Several procedures can detect and stage FHN presentation. Histological studies, scintigraphy, functional bone evaluations, radiography, magnetic resonance imaging (MRI), or computer-assisted tomography (CAT) are the most current diagnostic methods available.

Recent radiographic evaluation advancements allowed radiologists and orthopedic surgeons to identify FHN at earlier stages than previously possible [18]. Emerging technologies also prompted new management strategies that show different results according to the patient population and disease stage [43].

The most common differential diagnosis to FHN includes mid- to late-term osteoarthritis, acetabular dysplasia secondary osteoarthritis, ankylosing spondylitis involving the hip, idiopathic transient osteoporosis of the hip, chondroblastoma in the femoral head, subchondral insufficiency fracture, pigmented villonodular synovitis, synovial herniation, bone infarction and femoroacetabular impingement syndrome [44,45,46].

Once FHN diagnosis is correctly defined, there are several classification systems to stage it [47,48]. The first classification system developed for staging hip osteonecrosis by Ficat and Arlet was presented in the 1960s, followed by the Steinberg classification [49,50,51]. In 1991, the Association Research Circulation Osseous (ARCO) did recommend a third standardized classification system based on a comparison of different procedure findings to determine the size and location of the necrotic area: by radiograph, magnetic resonance imaging (MRI), computed tomography (CT), scintigraphy, and histologic findings [43]. Mitchell’s classification of avascular necrosis [52,53] is based on MRI signal characteristics within the center of the lesion on T1 and T2-weighted images. The Japanese Investigation Committee (JIC) classification [54], which has been adopted by the Japanese Ministry of Health, Labor, and Welfare (JMHLW), uses MRI images to classify osteonecrosis based on the location of the necrotic lesion relative to the acetabular weight; this bearing region may be an essential factor that determines the final prognosis [55,56,57,58]. To date, there is a lack of consensus on a universal and comprehensive system: choosing which one to use usually depends on the individual researcher’s preference. This lack of a standard classification is still a problem in the diagnosis and prognosis of FHN. The Ficat and Arlet system was the earliest yet remains the most widely used system. Newer classification systems have been developed, and some, such as the JIC, show promising prognostic value while maintaining simplicity. However, more extensive validating studies are needed [59,60]. Successful treatment depends on accurate staging; therefore, further multicenter collaborative efforts among osteonecrosis experts are required to adopt a universal classification system that may positively reflect patient outcomes.

Currently, FHN is diagnosed by plain anterior–posterior and frog-leg lateral radiographs of the hip, followed by magnetic resonance imaging (MRI). MRI is up to 100% sensitive for this diagnosis [61,62,63]. It is considered the gold standard: the presence of subchondral fracture suggests disease progression and may help define the treatment course. Computed tomography may be superior to MRI in detecting subchondral fracture [64,65], but further studies are necessary to determine if the additional cost and radiation exposure are justified. Other tools for assessing FHN presentation, such as bone marrow pressure measurements, venography, and core biopsy, are rarely used.

Unfortunately, to date, a unique algorithm for the treatment of FHN has not been defined. Surgery (joint-preserving and joint-replacing operations) is currently the most common approach. However, some patients receive conservative treatments consisting of pharmacologic agents and physical therapy, besides reducing physical exercise.

In general, conservative treatment of FHN may be useful, especially in the earlier stages of the disease. Although medical management can improve pain control, compliance, and functional outcomes, randomized clinical trials with long-term follow-up are necessary to determine therapy’s effectiveness.

A medical management program for FHN has been used increasingly during the early stages to hinder the disease’s progression. There are many potential medical therapies for FHN [66]. Lipid-lowering agents, anticoagulants, vasoactive substances, and bisphosphonates can all be used to manage FHN pharmacologically.

Increases in the number and size of circulating fat cells have been correlated with the development of FHN of the hip. Because of this, lipid-lowering agents such as statins are advantageous to reduce the rate of adipogenesis. Statins have been demonstrated to have a protective effect on patients receiving steroids [67,68,69].

Antiplatelets and anticoagulants such as enoxaparin and acetylsalicylic acid act via platelet aggregation inhibition, thus increasing blood flow to ischemic areas of the bone. These agents benefit patients with underlying coagulation disorders such as thrombophilia or hypo fibrinolysis [24,70,71,72].

Prostacyclin is a vasoactive agent that improves blood flow, an effect mediated through its vasodilator potential at the terminal vessel level. Its use has resulted in significant improvement in both clinical and radiologic outcomes of early-stage FHN. However, possible long-term benefits are still under evaluation. Other studies have evaluated vasodilators’ efficacy [73]; Iloprost represents an effective therapeutic option for treating bone marrow edema and FHN involving different locations in the body.

Bisphosphonates significantly decrease the incidence of collapse of the femoral head in osteonecrotic hips due to suppression of osteoclast activity. Alendronate has been used as adjunctive therapy in some procedures and has been found to reduce pain and the risk of collapse in the early stages of FHN [74,75,76,77,78,79].

Biophysical modalities described for treating FHN include extracorporeal shockwave therapy (ESWT), pulsed electromagnetic fields therapy (PEMF), and hyperbaric oxygen therapy (HBOT). ESWT [80,81] has been demonstrated to restore tissue oxygenation, reduce edema, and induce angiogenesis. ESWT can potentially offer a feasible and good substitute to more invasive surgical modalities currently used for FHN at its different stages. Though not widely used, PEMF is thought to function by stimulating osteogenesis and angiogenesis [82,83]. However, its role as early-stage FHN treatment remains to be established.

Hyperbaric oxygen therapy (HBOT) consists of breathing high oxygen concentrations at pressures that exceed 1 atm abs (101.325 kPa). Its efficacy is obtained by enhancing reactive oxygen species (ROS) and reactive nitrogen species (RNS) production, promoting cell growth, and modulating inflammatory response. As a result, vascularization and post-ischemic tissue survival is significantly improved [84,85,86]. ROS play a key role in many pathways leading to neoangiogenesis and vasculogenesis mediated by hypoxia-inducible factors (HIFs) and involving vascular endothelial growth factor (VEGF) and primary fibroblast growth factor (bFGF) [84,85,86].

In an in vitro reproduced chronic inflammatory model, HBOT improved the expression of osteogenic markers in mesenchymal stem cells (MSCs) and enhanced mineral deposition [87].

Treating osteoblasts with HBOT also improved their proliferation rate compared to hypoxic or normoxic conditions and increased alkaline phosphatase activity (ALP) [88]. HBOT promotes bone regeneration by improving osteosynthesis, neoangiogenesis, and vasculogenesis. HBO dissolved in the inflamed tissues concur to stimulate angiogenesis and enhance osteoclast and osteoblast functions of remodeling and repairing [85,86].

In pathologies characterized by osteonecroses, such as FHN and osteonecrosis of the knee [87,89,90], osteoradionecrosis, mandibular osteomyelitis, and dental implants, HBOT has been successfully used [84].

In patients with FHN, hyperbaric oxygen was demonstrated to reduce inflammatory markers such as tumor necrosis factor-α (TNF-α) and interleukin (IL)-6, creating a favorable osteogenic environment [90].

Hyperbaric oxygen therapy (HBOT) is a safe and effective option for patients with early stages of FHN. Radiographic improvements, reduced self-reported pain scores, increased range of motion, and improved patient’s quality of life is often reported early after the beginning of HBO treatments [26,91,92,93,94,95,96].

In addition to the clinical effects, HBO therapy could be a valid alternative to surgery in terms of associated costs [97,98,99,100]. Despite all these beneficial aspects, HBO treatments for FHN are still not approved worldwide. Therefore, this review aims to clarify the clinical effects of HBO therapy in treating FHN.

## 2. Materials and Methods

We conducted our systematic review in accordance with PRISMA guidelines.

### 2.1. Literature Search

A reviewer (EP), independently checked for accuracy by another (VZ), carried out a systematic literature search of the electronic databases PubMed, Cochrane Library, EMBASE, Virtual Health Library, and LILACS database from inception to 3 May 2020. This search used combinations of the following keywords: “Hyperbaric Oxygenation” OR “Hyperbaric Oxygenations” OR “Oxygenations, Hyperbaric” OR “Hyperbaric Oxygen Therapy” OR “Hyperbaric Oxygen Therapies” OR “Oxygen Therapies, Hyperbaric” OR “Oxygen Therapy, Hyperbaric” OR “Therapies, Hyperbaric Oxygen” OR “Therapy, Hyperbaric Oxygen” OR “Oxygenation, Hyperbaric.” The reference lists of the obtained studies were assessed by manual searching for any new eligible study. Titles and abstracts of potentially relevant articles were screened independently by EP and VZ. Full papers were screened for inclusion in this review if they fulfilled the following criteria: original research, adult participants (aged 16 years or older), contained a clear definition of FHN and HBO therapy. We did not include in this review editorials, commentaries, and government reports.

### 2.2. Eligibility Criteria

Two investigators reviewed the titles and abstracts (EP and VZ) to identify studies that met the following criteria: any human observational study (case–control, nested case–control, and cohort studies), patients with a definite diagnosis of FHN and HBO therapy and reporting of relative risk (RR) or odds ratio (OR) with 95% confidence interval (CI). If data from the same population were reported in more than one article, investigators included in the meta-analysis only the most recent, correct, and complete ones. Indeed, in the Chinese meta-analysis [91], we found several typing/calculation errors. Unfortunately, it was impossible to trace the sources of the individual studies included in it to assess their integrity. Therefore, we decided to use equally these data for the statistical analysis with the reserve and prospect to perform better data collection in future studies. Patient consent and institutional review board approval were not required for this meta-analysis.

### 2.3. Exclusion Criteria

In this meta-analysis, we applied the following exclusion criteria: animal studies, patients aged less than 16 years (due to different bone metabolism between young and adults), patients with a history of alcohol abuse or trauma to the involved hip, steroid use, patients treated with surgery, and patients with smaller lesions (such as transient osteoporosis or bone marrow edema alone).

### 2.4. Data Extraction

One investigator (EP) conducted data extraction and it was independently checked for accuracy by a second (VZ). For each included study, the following data were extracted: first author’s surname, publication year, country, study design, source of the study population, sample size, number of events/no events, range of ages, and adjusted RR/OR with 95% CI.

### 2.5. Statistical Analyses

Statistical analyses were conducted using Review Manager (RevMan [Computer Program] —version 5.4.0; The Cochrane Collaboration, 2020). An OR and 95% CI were used to analyze the statistical data. Χ2 tests evaluated the heterogeneity of the included articles. According to epidemiologic literature, when the study was statistically homogeneous (*p* > 0.1, I^2^ < 50%), a fixed-effects model was used for meta-analysis; if there was statistical heterogeneity among the studies (*p* < 0.1, I^2^ > 50%), the random-effects model was used. Subgroup and publication bias analyses were also conducted.

## 3. Results

Up to 3 May 2020, 10 studies [91,92,93,94] involving 353 controls and 368 HBO treated patients were included using the search strategy described. The total study number generated was 23,374. The review of the titles and abstracts, excluding duplicates and according to the inclusion and exclusion criteria, resulted in the exclusion of 23,314 articles. A full-text review of the remaining 60 items resulted in selecting the final ten studies, representing one American, one Israeli, one Taiwanese, and seven Chinese papers. All the studies were cohort studies.

**Figure 1:**
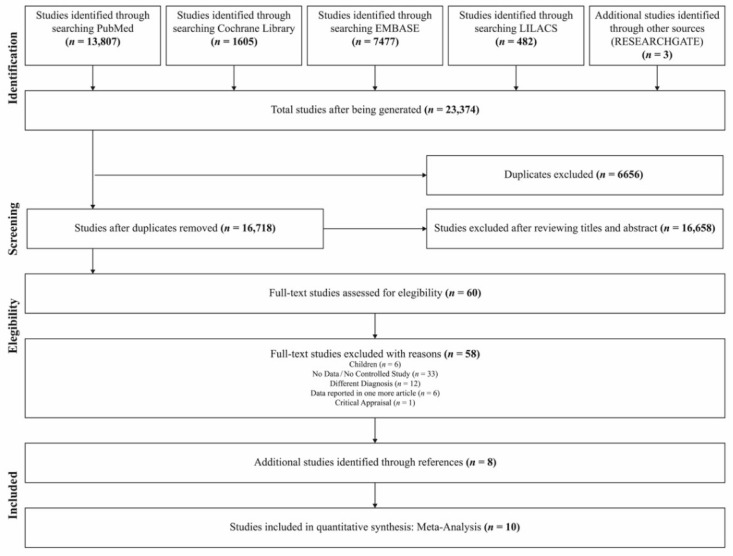
Flowchart of the selection strategy and inclusion/exclusion criteria used in the current meta-analysis.

**Figure 2:**
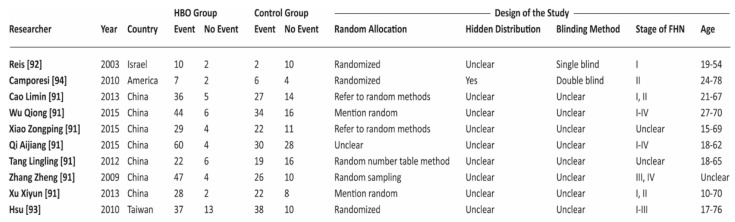
Characteristics and assessment of the methodological quality of trials included in the meta-analysis. Events = cases in which hyperbaric oxygen therapy (HBOT) was effective.

The search and exclusion process are shown in Figure 1, while in Figure 2, we report the general characteristics of the ten included studies. In the figure we reported as “events” the number of patients in which HBO was effective. Included studies adopted a quite uniform interpretation of efficacy of HBO therapy. In the different studies the positive outcome was evaluated both in terms of general clinical improvement (physical and mental relief, pain reduction, change in range of hip motion) and specific improvement at MRI.

**Figure 3:**
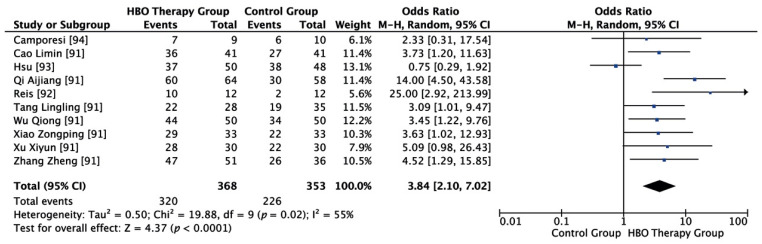
Evidence-supported HBO therapy in the treatment of femoral head necrosis. CI: confidence interval. M–H: Metropolis–Hastings. Events = cases in which HBOT was effective.

As shown in Figure 3, a total of 368 cases and 353 controls were included. Statistical analysis showed that I^2^ = 55%, *p* = 0.02. There is substantial heterogeneity, so the random effect model is adopted. Random effects model meta-analysis showed that clinical efficacy in the HBO group was 3.84 times higher than in the control group, and the difference was statistically significant (OR = 3.84, 95% CI (2.10, 7.02), *p* < 0.00001).

**Figure 4:**
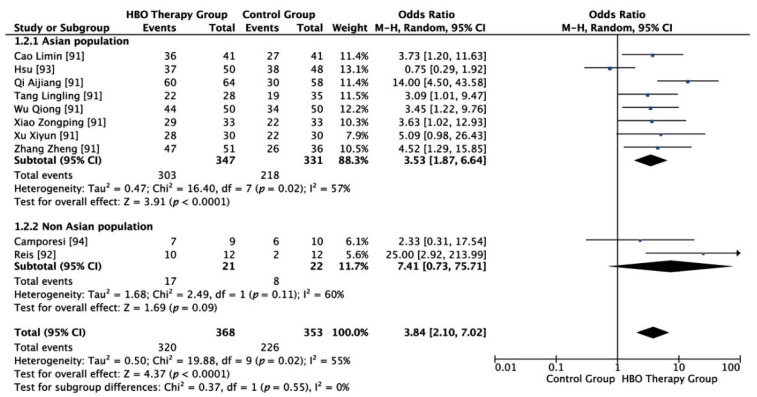
Evidence-supported HBO therapy in the treatment of femoral head necrosis (subgroup analysis). CI: confidence interval. M–H: Metropolis–Hastings. Events = cases in which HBOT was effective.

As shown in Figure 4, according to the subgroup analysis principle, the population was divided into Asian and non-Asian subpopulations. Among the included studies, eight refer to Asian populations and two to non-Asian populations. For the Asian subpopulation, random effect model meta-analysis showed that I^2^ = 57%, *p* = 0.02, and clinical efficacy in the HBO group was 3.53 times higher than in the control group, and the difference was statistically significant (OR = 3.53, 95% CI (1.87, 6.64), *p* < 0.00001). For the non-Asian subpopulation, random effect model meta-analysis showed that I^2^ = 60%, *p* = 0.11, and the clinical efficacy in the HBO group was 7.41 times higher than in the control group but the difference was not statistically significant (OR = 7.41, 95% CI (0.73, 75.71), *p* = 0.09).

**Figure 5:**
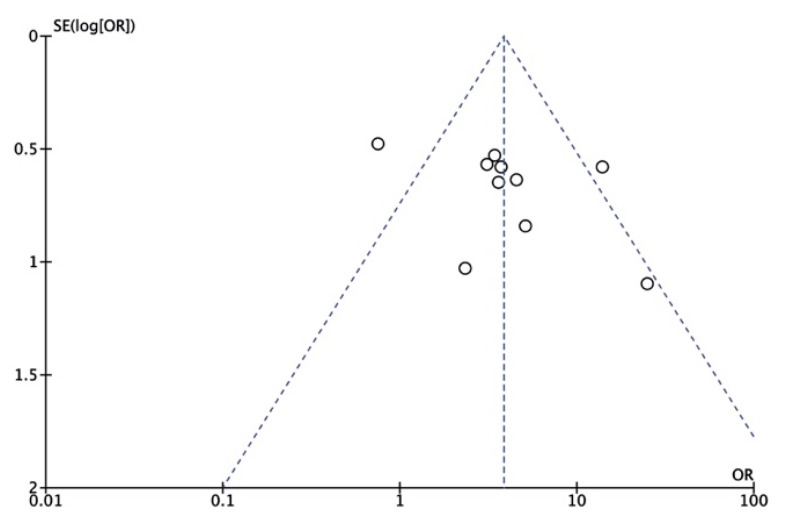
Funnel plot of publication bias. OR: odds ratio. SE: standard error.

**Figure 6:**
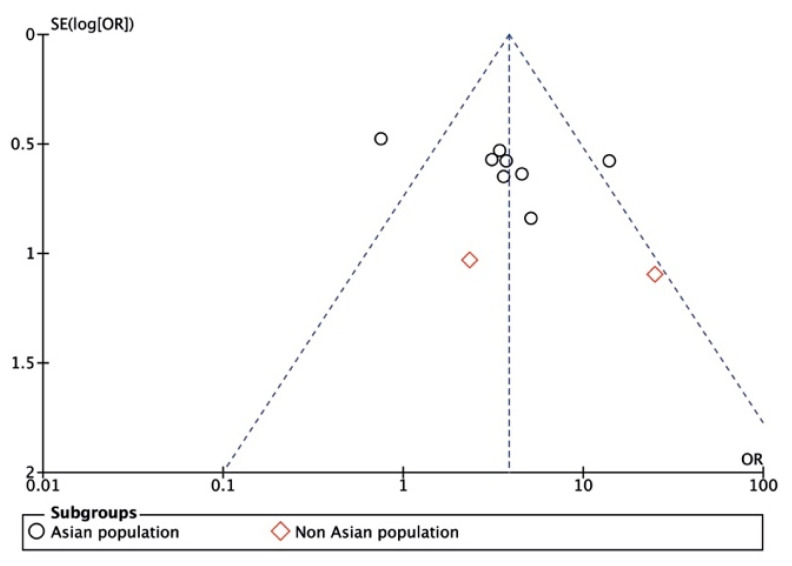
Funnel plot of publication bias (subgroup analysis). OR: odds ratio. SE: standard error.

As shown in Figure 5 and Figure 6, the results were in a symmetrical position, and the publication bias was neglectable.

## 4. Discussion

In 2016, at the Consensus Conference in Lille, FHN was accepted as an indication for hyperbaric oxygen therapy in the European Community level 2B [95]. Despite some clinical studies that support the benefits of HBO therapy in patients afflicted by osteonecrosis [26,94,95], this therapy is still not worldwide approved. Hence, this work aims to prove to extend the indication also for non-European countries.

HBO determines the elevation of the partial pressure of inspired O_2_ and the hydrostatic pressure. High O_2_ partial pressures lead to the increase in the production of reactive O_2_ species (ROS) and reactive nitrogen species (RNS) in various tissues [85]. HBO’s clinical efficacy depends on the modulation of intracellular transduction cascades, promoting the synthesis of growth factors and wound healing and reducing post-ischemic and post-inflammatory injuries [86,96]. The elevation of the hydrostatic pressure contributes to determining the compression of all gas-filled spaces in the body (Boyle’s law), and it is fundamental to allow effective treatment of those conditions where gas bubbles present in the body caused disease (e.g., decompression illness or intravascular) [26,101].

HBO therapy might also induce modulation of endothelial progenitor cell proliferation, promoting neoangiogenesis and neovascularization [102]. HBO increases extracellular oxygen concentration and reduces cellular ischemia and edema by causing vasoconstriction [103]. HBO reduces bone marrow pressure and improves oxygen delivery to ischemic cells, relieving compartment syndrome and preventing further necrosis.

Decreasing pressure induces significant pain relief. One of the first studies, proposed by Baixe and colleagues in 1969, stated that 20 HBO treatments were sufficient for pain reduction. Camporesi and colleagues showed that after 20–30 treatments, patients were substantially pain-free [94]. However, 20 HBO treatments are not considered sufficient for complete hip healing. Recent Italian work suggested a mean number of 83.3 ± 24.8 treatments [104]. In the study by Koren et al., the average number of treatments was 78.3 ± 24.2: this, in itself, is a remarkably close result [105].

Studies reported radiographic improvement in FHN Stage I according to the Steinberg classification. Additionally, they reported better pain control, compliance, and range of motion (ROM) in FHN at Ficat stages I–II [27,89,90,106,107]. Hyperbaric oxygen therapy also enhances osteoclast and osteoblast function for bone remodeling and repair. HBO also stimulates multi-potent fibroblasts in the bone marrow, aiding in osteogenesis, which is essential for bone tissue renovation [85,86,94,104].

Recent studies focusing on osteoblasts differentiation and suppression of osteoclasts activity show positive results derived from hyperbaric oxygen treatment. In particular, HBO shifted the balance between bone formation and bone resorption, promoting regeneration [88,108].

The osteoprotegerin (OPG)/Receptor Activator of Nuclear Factor κ-B (RANK)/Receptor activator of nuclear factor κ-Β ligand (RANKL) triad is the most important modulator of bone apposition/resorption balance [109]. Other controls of osteoclastic differentiation also exist, such as tumor necrosis factor-alpha (TNF-α), interleukin 6 (IL-6), and interleukin 1 (IL-1). These cytokines can modulate the triad’s biological activities that interfere directly with RANK–RANKL binding signaling pathways and induce osteoclast differentiation and activation [110,111,112,113]. Many studies have already investigated the anti-inflammatory effect of HBO on many diseases [114,115,116]. As a consequence, this effect is also suggested for FHN. Indeed, OPG/RANKL/RANK may be considered an osteo-immunomodulator complex. HBO may act in modulating not only OPG but also pro-inflammatory cytokines in an “anti-osteoclastic” manner.

Hyperbaric oxygen therapy also increases the production of reactive oxygen species (ROS) and reactive nitrogen species (RNS) in various tissues [85]. Several studies compare non-traumatic FHNs with controls by analyzing different gene polymorphisms of endothelial nitric oxide synthase (eNOS). They show how some polymorphisms of this enzyme can be involved in the etiology of FHN [117,118,119,120,121,122]. Nitric oxide’s (NO) decreased production promotes the recruitment, aggregation, and adhesion of platelets that compromise angiogenesis and bone formation. The eNOS polymorphism demonstrates a multidirectional cause of FHN. The eNOS polymorphism may be an independent cause of idiopathic osteonecrosis and maybe a synergistic cause of secondary FHN with other etiopathologies of osteonecrosis.

Further studies are needed to establish the relationship between the eNOS polymorphisms and idiopathic or secondary FHN. A meta-analysis [122] supports the hypothesis that single-nucleotide polymorphisms (SNPs) in vascular endothelial growth factor (VEGF), eNOS, and ATP-binding cassette subfamily B member one transporter (ABCB1) are independent risk factors for the development of FHN. VEGF is an essential molecule in angiogenesis and plays a vital role in bone formation and repair (normal growth plate morphogenesis, including blood vessel invasion and cartilage remodeling). eNOS, the main NOS isoform expressed in bone, modulates bone resorption and osteoclast formation. Additionally, the anabolic effects of estrogen and insulin-like growth factor depend, in part, on the NO produced by eNOS; ABCB1 encodes P-Glycoprotein (P-gp), which is involved in the active transport of corticosteroids and is linked to the development of FHN, potentially affecting steroid metabolism. However, further large-scale studies of patients are needed to confirm these findings. A study’s results indicate that EGF promotes bone formation and micro vascularization in FHN and positively affects femoral head preservation [123].

Thanks to these beneficial effects, in Italy, femoral head necrosis is included in accepted indications supported by reimbursement agencies. Its treatment protocol consists of one treatment/day, five to six days/week, ≥60 min between 2.2–2.5 Atmospheres Absolute (ATA), with FiO_2_ = 1, for 60 to 90 treatments. The total number of treatments (60 to 90) suggested for these cases, according to the Italian guidelines for HBO therapy, is close to the gross average number of treatments in different studies analyzed (70 treatments) [92,93,94,96,105].

In particular, a recent study [106] suggests as appropriate some daily treatment of ≥60 min at FiO_2_ = 1 (5 to 6 days a week, and 4–5 weeks per cycle) at 2.4 ± 2.5 ATA, at the initial stage of FHN (Type 2 recommendation; Level B evidence) or 60–90 HBO_2_ treatments at the initial stage of FHN (Type 3 recommendation; Level C evidence), and suggests to schedule MRI and orthopedic clinical evaluation at 3–4 weeks from the end of the HBO cycle (Type 2 recommendation; Level C evidence). It suggests that it could be reasonable to adopt different measures and strategies to make recovery faster: to reduce weight-bearing exercise (use crutch suitable for the height of the patient and contralateral to the lesion), to reduce Body Mass Index (BMI), to do physical therapies if applicable, and quit smoking (smoke reduces the efficacy of the treatment) (Type 1 recommendation; Level C evidence).

Therefore, there are numerous physiological and pharmacological benefits to HBO therapy: reduction in the edematous component of a lesion, better tissue oxygenation, and the possibility of restoring venous drainage both recovering the affected bone district (due to a progressive and sharp decrease in the intraosseous pressure) and improved local microcirculation (thanks to increased angiogenesis) [85,86] and modulation of bone formation/resorption promoting regeneration [88,108].

In addition to the clinical effects, HBO therapy could be a valid alternative to surgery in terms of the impact cost [97,98,99]. Similar to all pathologies, FHN has direct and indirect costs. There are also intangible costs that concern more subjective and psychological aspects that are very difficult to evaluate and quantify (pain, decreased quality of life, mood alteration, and impairment in physical activities).

Considering that the average reimbursement charge usually applied in Italy (currently 1.08 Euros per US Dollar) for a single treatment is about 100.00€ and the total number of treatments suggested for FHN, according to the Italian guidelines, are 60 to 90, the average cost per patient appears to range from $6503.16 to $9754.74 US. This cost appears reasonable compared to the amount to the direct and indirect costs related to one or more surgical interventions and rehabilitation. In Italy, total hip arthroplasty (THA) costs about 10,000.00€ (10,838.60$) (DRG 544), THA reoperation (DRG 545) costs about 12,000.00€ (13,006.32$), and then, after each surgery, there is the rehabilitation that costs 250.00 €/day (270.97 $/day).

Patients often need more interventions because hip prostheses deteriorate and wear out during the years. There are not enough pieces of information yet available to calculate precisely how long a hip prosthesis could last. A recent meta-analysis [97] estimates that about three-quarters of hip replacements survive 15–20 years and only over half last 25 years in patients with osteoarthritis. Post-operative complications after THA surgery can require multiple treatments, including implant removal and reimplantation [98]. Therefore, THA costs remain high [99]. Cost control measures such as centralizing common, costly surgeries, such as THA, to high-volume centers of excellence enhanced patient outcomes, but costs have not dropped [100]. Cost considerations and efficacy suggest HBO therapy as a valid alternative.

The current meta-analysis results indicate that HBO therapy in the treatment of femoral head necrosis can significantly improve patients’ clinical outcomes through its therapeutic and pleiotropic effects. Both Asian and non-Asian populations can benefit from this method.

The protocol has not been registered in PROSPERO since it does not accept students’ reviews and the article was first conceived as a master’s degree thesis.

The limitations of this meta-analysis derive mostly from the constraints imposed by the quantity and quality of research. It is necessary to carry out a large-sample randomized controlled trial in the future. Therefore, this work suggests that future clinical trials should pay attention to several aspects and hypothesized needs for better evidence:

1. Design a standard for defining the most proper characteristics in patients’ selection (age, race, BMI, clinical and pharmacological history, stage of FHN by a unique method of classification, unilateral or bilateral hips involved, use or not of crutch suitable, smoke or not).
2. Standardize the HBO dives for the proposed indication (ATA, time, number of treatments).
3. Define and standardize the best follow up protocol to adopt.
4. Adopt a shared standard for data presentation (number of the hips treated and not of patients).
5. Long-term timing in the study follow-up to facilitate observation of the endpoint to draw a positive conclusion.

## 5. Conclusions

The results of this meta-analysis suggest that patients with femoral head necrosis treated by HBO therapy at early stages can achieve a significantly improved clinical treatment effect, in particular the Asian population showed statistically significant results, probably because of a superior numerosity.

## Data Availability

Data and details are contained within the article.
